# Two-Staged Implant-Based Breast Reconstruction: A Long-Term Outcome Study in a Young Population

**DOI:** 10.3390/medicina55080481

**Published:** 2019-08-14

**Authors:** Oscar J. Manrique, Ali Charafeddine, Amjed Abu-Ghname, Joseph Banuelos, Steven R. Jacobson, Jorys Martinez-Jorge, Minh-Doan Nguyen, Christin Harless, Nho V. Tran, Basel Sharaf, James W. Jakub, Tina J. Hieken, Amy C. Degnim, Judy C. Boughey

**Affiliations:** 1 Division of Plastic Surgery, Mayo Clinic, Rochester, MN 55904, USA; 2 Department of Surgery, Mayo Clinic, Rochester, MN 55904, USA

**Keywords:** implants-based breast reconstruction, young females, reconstructive outcome, adjuvant radiation, obesity

## Abstract

*Background and objectives:* Differences in patient anatomy and physiology exist between young and older patients undergoing breast reconstruction after mastectomy. Breast cancer has been described as being more aggressive, more likely to receive radiation, contralateral mastectomy, as well as bilateral reconstruction in young patients. Our purpose is to report long-term experience on two-staged implant-based breast reconstruction (IBR) in young females, with complication sub-analysis based on obesity and adjuvant radiation. *Materials and Methods:* Retrospective chart review of all consecutive young patients who underwent two-staged IBR at our institution, between 2000 and 2016, was performed. Patients between 15 and 40 years old with least 1-year follow-up were included. Univariate logistic regression models and receiver operating characteristic (ROC) curves were created. *Results:* Overall 594 breasts met our inclusion criteria. The mean age was 34 years, and the median follow-up was 29.6 months. Final IBR was achieved in 98% of breasts. Overall, 12% of breasts had complications, leading to explantations of 5% of the devices. Adjuvant radiation was followed by higher rates of total device explantations (*p* = 0.003), while obese patients had higher rates of total complications (*p* < 0.001). For each point increase in BMI, the odds of developing complications increased 8.1% (*p* < 0.001); the cutoff BMI to predict higher complications was 24.81 kg/m^2^. *Conclusions:* This population demonstrates high successful IBR completion and low explantation rates. These data suggest that obese women and those with planned adjuvant radiation deserve special counseling about their higher risk of complications.

## 1. Introduction

Breast reconstruction following mastectomy has been shown to improve the quality of life as well as the social and psychological satisfaction of female patients undergoing mastectomy [1,2,3,4] Several options exist to reconstruct the breast such as autologous, implant-based reconstruction or a combination of both [5,6]. Implant-based breast reconstruction (IBR) remains the most common reconstructive approach in the United States and worldwide among women undergoing mastectomy [7,8,9,10]. According to data from the American Society of Plastic Surgeons, it is estimated that more than 106,000 implant-based reconstructive procedures were performed in 2017. Regardless of the technique used, it is important to acknowledge that breast reconstruction is an integral component of breast cancer care, as it improves the quality of life and alleviates the psychological trauma of breast cancer patients [2,11].

Increased awareness and uptake of panel genetic testing, as well as advanced screening protocols, have led to an increase in the number of young patients undergoing mastectomy with subsequent reconstruction [12,13] Medical and surgical treatments in addition to reconstruction of the breast in this group of patients remain challenging. Younger patients, often defined as younger than the age of 40, tend to have more aggressive cancer with higher recurrence rates [14,15,16,17,18]. These patients are also more likely to have an underlying genetic predisposition and an increased risk of developing a second primary tumor [19,20,21,22,23]. Furthermore, reconstruction is challenging, as current data suggests that psychosocial distress, self-image and sexuality are more negatively affected in this young population [24]. Younger patients are also more likely to choose IBR due to concerns over donor site morbidity associated with autologous reconstruction, potential effect on future pregnancies, and the greater likelihood of undergoing a contralateral mastectomy [25,26,27]. Regardless of a patient’s age, IBR is associated with a number of complications and certain risks that may affect the final aesthetic outcome [28,29,30,31,32,33]. The associated risk factors include radiation, obesity, diabetes and bilateral reconstruction. Adjuvant radiation and obesity is particularly important in young women due to an increased risk of local recurrence and the growing obesity epidemic [34,35,36,37]. 

A limited number of studies have assessed the outcomes and complications of IBR in this young group of patients [27,38,39]. Herein, we present our institution’s experience with two-staged, implant-based breast reconstruction, whether immediate or delayed, following either prophylactic or therapeutic mastectomy in patients younger than 40 years of age. 

## 2. Materials and Methods

After approval by our Institutional Review Board (IRB No. 17-002553; from May 1st, 2017), a retrospective electronic chart review was performed to identify all consecutive patients who underwent IBR, from May 2000 to September 2016. Inclusion criteria encompassed patients between the ages of 15 and 40 years old who had both their mastectomy and IBR performed at our institution. Patients with less than 12 months of follow up, with incomplete charts, or with planned autologous reconstruction, were excluded.

Patient demographics were collected, including age, body mass index (BMI), smoking status, comorbidities, type of mastectomy (skin sparing, nipple sparing and total), intention of surgery (therapeutic or prophylactic), breast pathology (benign, in situ or invasive), cancer stage, type of reconstruction (immediate or delayed), location of the implant (sub-pectoral or pre-pectoral), use of acellular dermal matrix (ADM) and oncologic treatment (radiation and/or chemotherapy). All cases of adjuvant radiation occurred during expansion, prior to the implant exchange. The intention of surgery was considered therapeutic for patients with in situ and invasive malignancies, and prophylactic in patients with a high risk of cancer but with benign breast pathology reports. Follow-up time was recorded in months for each patient.

Postoperative complications and outcomes were reviewed using predetermined definitions and a manual chart review. For the purposes of this study, primary wound dehiscence was defined as full-thickness wound separation unrelated to preceding complications. Skin flap necrosis was defined as full-thickness skin necrosis. Seroma and hematomas were defined as those that were symptomatic and required aspiration or evacuation in the operating room. Breast implant infection was defined using the Centers for Disease Control and Prevention (CDC) criteria for Surgical Site Infections (SSI) [40]. Device deflation was defined as cases of spontaneous implant or Tissue Expander (TE) deflation or rupture. Implant and TE explantation were defined as device removal secondary to a complication. Failed reconstruction was considered when IBR was abandoned, and autologous reconstruction was pursued instead.

### Statistical Analysis

Continuous data was evaluated by the Shapiro-Wilk test for normal distributions. Data with normal distribution was reported as means with standard deviation and comparisons were performed with the t-test. Data with no normal distribution was presented as medians with interquartile ranges for the 25th to 75th percentile, and comparisons were performed with the Mann-Whitney-Wilcoxon test. Categorical data was presented as a percentage, and analyzed using the chi square test, and for small samples (less than five units) the Fisher’s exact test was used. Univariate logistic regression models were performed to assess the effect of adjuvant radiation and obesity on surgical complication. For a sub-analysis in obese patients, breasts were divided into those with BMI = 30–34.9 kg/m^2^ and those with BMI ≥ 35 kg/m^2^. Additionally, receiver operating characteristic (ROC) curves were created to identify BMI cutoff points for increased risk of complications. Statistical analysis was performed using STATA 16.0 software.

## 3. Results

### 3.1. Demographics

Between May 2000 and September 2016, a total of 315 consecutive patients younger than the age of 40, including 594 breasts, underwent two-staged IBR at our institution. Of those, 279 patients (88.5%) had bilateral reconstructions. The average age was 34.2 years and ranged between 15 to 39 years. Median follow-up time was 29.6 (interquartile range [IQR]; 17–60) months. Patient demographic characteristics are summarized in Table 1. The mean BMI was 25.3 (±5.8) and 54 (17%) patients had a BMI ≥ 30. Eighteen (6%) patients had one or more comorbidities. History of smoking was reported in 52 patients (16.5%).

### 3.2. Cancer and Reconstructive Modalities

The most common type of mastectomy was skin sparing mastectomy in 335 (56.4%), and 247 (41.6%) had nipple sparing mastectomy. The mastectomy was prophylactic in 59.4% of the breasts and therapeutic in the remaining 40.6%, with invasive pathology reported in 199 (33.5%) of the mastectomies. Clinical characteristics are shown for the patients; 6.3% had delayed reconstructions. The majority of them in Table 2. Immediate breast reconstruction was the most common pathway of reconstruction, and 93.7% of reconstructions were sub-pectoral (70.7%). Overall, ADM was used in 464 (78.1%) breasts. Prior to the reconstruction, 7 (1.2%) breasts had a history of radiation. 

Eighty (25.3%) patients received neoadjuvant chemotherapy, while 116 women (36.8%) received adjuvant chemotherapy. Seventy-Five (12.6%) reconstructed breast mounds received adjuvant radiotherapy.

### 3.3. Reconstructive Outcomes

Overall, 72 (12%) breasts had complications. Outcomes and complication rates are shown in Table 3. Surgical complications occurred more frequently during the first stage (9.2%) than the second stage (3.9%; *p* < 0.001). First stage complications included: 12 (2%) Seromas, 6 (1%) hematomas, 13 (2.2%) with primary wound dehiscence, 10 (1.7%) skin flap necroses, 18 (3%) breast implant infections, and 7 (1.2%) deflations/ruptures. Conversely, 23 (3.9%) complications occurred after the second stage, including: 3 (0.5%) Seromas, 2 (0.3%) hematomas, 6 (1%) with primary wound dehiscence, 3 (0.5%) skin flap necroses, 7 (1.2%) breast implant infections, and 2 (0.3%) implant ruptures. Finally, long-term complications such as capsular contracture affected 14 (2.4%) breasts during the follow-up period of the study.

Thirty (5%) devices (tissue expanders and final implants) had to be explanted secondary to a complication. Of these, 20 (4.3%) tissue expanders were explanted after the first stage procedure due to the following reasons: nine infections, seven tissue expander deflations, two concerning primary wound dehiscence, and two skin flap necroses. Following the second stage procedure, 10 (1.7%) implants were explanted; six due to infections, two due to wound dehiscence, one due to skin necrosis, and one due to implant rupture. In conclusion, following the 30 device explantations, 17 breasts successfully underwent device exchange, while 13 (nine tissue expanders and four implants) underwent autologous-based breast reconstruction. Out of the 594 breasts, final implant-based breast reconstruction was completed in 581 (97.9%) breasts, while in the remaining 13 (2.1%) breasts, implant-based reconstruction was abandoned, and autologous reconstruction was performed instead. 

### 3.4. Effect of Adjuvant Radiation and Obesity

An analysis of the specific effect of obesity and adjuvant radiation following mastectomy on complication rates was performed. Table 4 demonstrates the association of adjuvant radiation with increased risk of complications. Patients with adjuvant radiotherapy had higher complication rates during their first stage (12% vs. 8.8%), second stage (7% vs. 3.5%), and in total reconstruction (17% vs. 11.4%); however, these differences were not statistically significant (*p* = 0.158, *p* = 0.179, and *p* = 0.139, respectively). On the other hand, patients with radiation had significantly higher rates of implant explantation (5.6% vs. 1.2%) and total device explantation (12% vs. 4%) (*p* = 0.024, and *p* = 0.003, respectively).

Patients with obesity demonstrated significantly higher rates of complications during the first stage (17.8% vs. 7.5%; *p* < 0.001), second stage (9.9% vs. 2.7%; *p* < 0.001), and in overall reconstruction (24.7% vs. 9.5%; *p* < 0.001, see Table 5). For each point increase in BMI, the odds of developing complications increase by 8.5% (*p* < 0.001) and based on the ROC curve, the cutoff BMI to predict higher complications rates during reconstructions was 24.81 kg/m^2^. 

These higher rates were particularly evident in breast implant infection (9% vs. 3.2%), seroma (7% vs. 1.6%), skin flap necrosis (6% vs. 1.4%), and wound dehiscence (5% vs. 2.8%). Rates of tissue expander (4.9% vs. 3.0%; *p* = 0.332), implant (3.9% vs. 1.2%; *p* = 0.067), and total device explantations (8.9% vs. 4.3%; *p* = 0.052) were numerically, but not statistically, significantly, higher in obese patients.

Obese patients were further subdivided into two groups, those with BMI between 30–35 and those with BMI ≥ 35. Patients with BMI ≥ 35 demonstrated significantly higher rates of first stage procedure complications (30.7% vs. 12.9%; *p* = 0.028) and tissue expander explantations (15.4% vs. 1.6%; *p* = 0.008) as well as total reconstruction complications (41.0% vs. 17.7%; *p* = 0.010) and device explantations (23.1% vs. 3.2%; *p* = 0.002). Outcomes and complications of obese patient’s subgroups are summarized in Table 6. 

## 4. Discussion

Two-staged implant-based breast reconstruction is feasible with excellent outcomes in females under the age of 40. Our study, evaluating 594 mastectomies with two-staged implant-based reconstruction in 315 women younger than the age of 40, found that final IBR was successful in 98%, with a low overall complication rate of 12% during the study period. These complications lead to a total device explantation rate of 5%. Our analysis showed that obese patients had significantly higher rates of first stage, second stage and total reconstruction complications compared to non-obese patients. In addition, breasts that underwent radiation had significantly higher rates of implant and total device explantations.

Prophylactic mastectomy is an option for women at a high risk of developing breast cancer, with the highest risk reduction occurring if undertaken before the age of 40 years old [41,42]. As a result, young women are more likely to undergo prophylactic mastectomy and bilateral reconstruction [25]. Breast cancer generally affects older patients, with only a small percentage affecting women under the age of 40 [43,44,45]. 

Young patients who elect to have reconstruction represent a challenge, as the biology of the tumor is often more aggressive, and patients are more likely to undergo IBR [27,46]. The ultimate choice of reconstruction is based upon several factors such as age, tumor grade and stage, patient desire and adjuvant therapy. In our patients’ cohort, 36% of the patients received adjuvant chemotherapy and 12.6% of the breasts required post-operative radiotherapy (Table 2). Adjuvant oncologic treatment rates are lower than those reported by Vogel et al. in 2011 in a similar younger population [27]. Their study reported an adjuvant chemotherapy rate of 66% and radiotherapy of 29.2%.

Implant-based reconstruction has significantly evolved over the last two decades [7,8,9,10]. While historically, muscle coverage has been advocated as the preferred reconstructive approach, current practices have evolved towards pre-pectoral implant reconstruction [47,48,49,50,51,52,53,54,55,56,57]. The pre-pectoral approach re-emerged as a valuable reconstructive technique following the recent technological advances such as ADM, and intraoperative flap perfusion technology [58,59,60,61,62]. Due to the time period of this study, 71% of the implants were placed sub-pectorally, and only 29% were pre-pectoral implants, as pre-pectoral IBR became the most common reconstructive approach in our institution in 2014. Due to the small sample size of pre-pectoral reconstruction in this cohort, as well the wide variation in surgical technology and operating room personnel between the two groups in the long study period, we were not able to compare pre-pectoral versus sub-pectoral reconstruction.

In a study done by Matsumoto et al. breasts were divided into three age groups based on the World Health Organization (WHO) classifications [38]. Out of the 153 patients who were in the young group, 63 (41%) had complications [38]. However, in their study, complications were not stratified based on the reconstructive approach, whether implant-based or autologous, and only 51% of their breast reconstructions were implant-based. Furthermore, in 2016 Santosa et al. reported a complication rate of 21.5% and a failure rate of 5.9% in 373 IBRs in women younger than 45 years old [39]. In our larger cohort study, we noted a lower total complication rate of 12% and device explantation rate of 5%, with comparable follow-up time to previously published studies.

Using previously published data on IBR complications in the general population as a comparison, our younger population results show comparable complication rates [63,64,65,66]. In a systematic review published by Georgetown University Hospital in 2013, analysis of 679 two-staged IBRs demonstrated a pooled overall complication rate of 52.8% [67]. In a different retrospective review by Spear et al. of 428 two-staged IBRs, the incidence of device explantations secondary to complications was 3.5% in the first stage and 1.9% in the second stage. Our study demonstrated similar rates of 4.3% and 1.7%, respectively, with a longer follow up period [64]. In addition, our study showed lower capsular contracture rates than previously reported, which may in part be explained by the higher proportion of sub-pectoral implants and prophylactic mastectomies in our population [64].

Adjuvant radiation is especially important for young women with invasive breast cancer, as their absolute risk of local recurrence is higher than for older women [36,37]. As a result there is even a greater benefit from adjuvant radiation [36,37]. Reconstructive challenges and complications that accompany breast radiation are well documented in the literature [68,69]. Our analysis demonstrated that reconstructed breast mounds that underwent radiotherapy had significantly higher associations with implant (5.6%) and total device explantation (12%) rates. The increased risk of complications associated with radiotherapy was also established by Spear et al., with an implant failure rate of 10% in irradiated breasts. Despite this significant association, device explantations in irradiated breasts occurred less frequently than previously reported in literature [70,71]

Our analysis revealed that obese patients were significantly more likely to suffer first stage, second stage and total reconstruction complications. Obesity is defined as having a BMI ≥ 30 kg/m^2^ and currently 1 in 3 individuals living in the United States are obese [34,35]. In our young population, 17% of the patients were obese. Obese patients are susceptible to decreased wound healing due to impaired immunity and elevated blood sugar levels [72,73]. 

In addition, this particular population poses a challenge due to longer operative time and technical difficulties [74]. Our findings comply with previous studies on the association between obesity and increased risk of postoperative complications, including seroma, infection and reconstructive failure [32,74,75,76,77]. In addition, our analysis showed that the odds of complications increased 8.1% for each unit increase in a patient’s BMI. This was further supported by our sub-analysis in obese patients that demonstrated significantly higher rates of overall complications and device explantations in those with BMI ≥ 35 compared to those with BMI = 30–34.9.

### Limitations and Recommendations

This study is a large case series with a long duration and a relatively long follow-up time that focus solely on the surgical outcomes of two-staged, implant-based breast reconstruction in young patients; however, it has certain limitations. It is a single-institution retrospective study with the potential for surgical bias. The long study period encompassed a large number of surgeons and different surgical techniques and implants, and materials technologies, as well as inconsistent reporting of certain pertinent variables, such as *BRCA* mutations. Furthermore, numerous parents were lost to follow-up after one year, and as such this limited a longer follow-up period. Collectively, these limitations hindered our ability to include an older control group for a more robust analysis and stronger conclusions. Important issues such as cost and cosmetic outcomes were not addressed, as our study was limited to reporting surgical outcomes and complications. In addition, timing of the adjuvant radiotherapy in relation to the stage of breast reconstruction was not taken into consideration.

## 5. Conclusions

The outcomes of our study show that implant-based reconstruction is an effective and a safe approach in young women. This population demonstrated high total implant-based reconstruction completion and low device explantation rates. These data suggest that despite their young age, obese women and those with planned post-operative radiation deserve special counseling about their higher risk of complications. New strategies to address the best methods for reconstruction in these patient populations are still needed.

## Figures and Tables

**Table 1:** Patient demographics.

Variable	Total, *n* (%)
Total patients	315
Total breasts	594
Age *	34.2 ± 4.3
Laterality Bilateral Unilateral	279 (88.5) 36 (11.5)
BMI * Obese	25.3 ± 5.8 54 (17%)
History of Smoking	52 (16.5)
Comorbidities	
DM	5 (1.6)
HTN	15 (4.7)
Follow-up, months ^±^	29.6 (17–60)

BMI: Body mass index, DM: Diabetes mellitus, HTN: Hypertension, * Mean (± standard deviation), ^±^ Median (IQR: 25–75%).

**Table 2:** Clinical characteristics.

Characteristics	Total, *n* (%)
Pathology	
Benign pathology	353 (59.4)
In situ malignancy	42 (7.0)
Stage I	72 (12.1)
Stage II	89 (15.0)
Stage III	35 (6.0)
Stage VI	3 (0.5)
Intention of mastectomy	
Prophylactic	353 (59.4)
Therapeutic	241 (40.6)
Mastectomy type	
Nipple sparing	247 (41.6)
Skin sparing	335 (56.4)
Total	12 (2.0)
Type of reconstruction	
Immediate	557 (94.7)
Delayed	37 (6.3)
ADM use	464 (78.1)
Location of implant	
Subpectoral	420 (70.7)
Prepectoral	174 (29.3)
Oncologic treatment	
Chemotherapy *	
Neoadjuvant	80 (25.3)
Adjuvant	116 (36.8)
Radiotherapy	75 (12.6)
History of radiation	7 (1.2)

DTI: Direct to implant, ADM: Acellular dermal matrix. * Reported per patient.

**Table 3:** Surgical site complications and outcomes.

Complication	1st Stage * *N*, (%)	2nd stage * *N*, (%)	Total Reconstruction * *N*, (%)
No. of breasts	594	583	594
Seroma	12 (2.0)	3 (0.5)	15 (2.5)
Hematoma	6 (1.0)	2 (0.3)	8 (1.3)
Wound dehiscence	13 (2.2)	6 (1.0)	19 (3.1)
Skin flap necrosis	10 (1.7)	3 (0.5)	13 (2.2)
Breast implant infection	18 (3.0)	7 (1.2)	25 (4.2)
Device deflation	7 (1.2)	2 (0.3)	9 (1.5)
Total Complications	55 (9.2)	23 (3.9)	72 (12.1)
Device Explantation	26 (4.3)	10 (1.7)	36 (6.0)
Failed reconstruction	9 (1.5)	4 (0.7)	13 (2.1)

* Number of breasts that encountered complications.

**Table 4:** Outcomes Analysis for Adjuvant Radiation.

	With Radiation *N*, (%)	Without Radiation *N*, (%)	*p*
No. of breasts	75	519	
1st stage complications	9 (12.0)	46 (8.8)	0.381
TE explantation	5 (6.7)	15 (2.9)	0.158
2nd stage complications	5 (6.7)	18 (3.5)	0.179
Implant explantation	4 (5.6)	6 (1.2)	0.024
Total reconstruction complications	13 (17.3)	59 (11.4)	0.139
Total device explantation	9 (12.0)	21 (4.0)	0.003

TE: Tissue expander.

**Table 5:** Outcomes analysis for obesity.

	Obese *N*, (%)	Non-Obese *N*, (%)	*p*
No. of breasts	101	493	
1st stage complications	18 (17.8)	37 (7.5)	<0.001
TE explantation	5 (4.9)	15 (3.0)	0.332
2nd stage complications	10 (9.9)	13 (2.7)	<0.001
Implant explantation	4 (3.9)	6 (1.2)	0.067
Total reconstruction complications	25 (24.7)	47 (9.5)	<0.001
Total device explantation	9 (8.9)	21 (4.3)	0.052

**Table 6:** Stratified analysis for obese patients.

	BMI 30–34.9 *N*, (%)	BMI ≥ 35 *N*, (%)	*p*
No. of breasts	62	39	
1st stage complications	8 (12.9)	12 (30.7)	0.028
TE explantation	1 (1.6)	6 (15.4)	0.008
2nd stage complications	5 (8.1)	5 (14.3)	0.488
Implant explantation	1 (1.6)	3 (8.6)	0.132
Total reconstruction complications	11 (17.7)	16 (41.0)	0.010
Total devices explantation	2 (3.2)	9 (23.1)	0.002
